# Supplementary material for: Linear and Machine Learning modelling for spatiotemporal disease predictions: Force-of-Infection of Chagas disease
Source: PLoS Negl Trop Dis. 2022 Jul 19;16(7):e0010594. doi: 10.1371/journal.pntd.0010594 (PMC9337653; doi:10.1371/journal.pntd.0010594)
Supplement: S1 Appendix — (DOCX) [file pntd.0010594.s001.docx]

**Linear and Machine Learning Modelling for Spatiotemporal Disease Predictions: Force-of-Infection of Chagas Disease**

**Julia Ledien^1^, Zulma M. Cucunubá^2,3^, Gabriel Parra-Henao^4,5^, Eliana Rodríguez-Monguí^6^, Andrew P. Dobson^7^, Susana B. Adamo^8^, María-Gloria Basáñez^2^, Pierre Nouvellet^1^**

¹School of Life Sciences, University of Sussex, Falmer, Brighton, UK

²London Centre for Neglected Tropical Disease Research & MRC Centre for Global Infectious Disease Analysis, School of Public Health, Imperial College London, London, UK

^3^Departamento de Epidemiología Clínica y Bioestadística, Facultad de Medicina, Universidad Pontificia Javeriana, Bogotá, Colombia

^4^Centro de Investigación en Salud para el Trópico, Universidad Cooperativa de Colombia, Santa Marta, Colombia

^5^National Institute of Health, Bogotá, Colombia

^6^ Independent consultant to the Neglected, Tropical and Vector Borne Diseases Program, Pan American Health Organization (PAHO), Colombia

^7^Department of Ecology and Evolutionary Biology, Princeton University, Princeton, New Jersey, USA

^8^Center for International Earth Science Information Network (CIESIN), The Earth Institute, Columbia Climate School, Columbia University, New York, USA

Corresponding author: Julia Ledien, School of Life Sciences, University of Sussex, UK, [j.ledien@sussex.ac.uk](mailto:j.ledien@sussex.ac.uk)

**S1_Appendix: Machine Learning tuning**

- - 1. Resampling strategies

Spatial only, temporal only and spatiotemporal resampling strategies were tested and compared to a standard random resampling strategy. Also, the resampling strategy that was developed for the linear framework (which consisted of selecting one value (i.e. year) for each serosurvey at each iteration) was tested (Table 1). Values of between 5 and 50 folds (number of subsets the data are divided into) were tested and eventually set at 10 as no substantial changes were observed on the performance indicators.

Table 1: Resampling strategies tested

| Model Name | Parameters used | Task type | Resampling method |
| --- | --- | --- | --- |
| Random | default parameters | default | Random 10 folds |
| Temporal | default parameters | temporal | LTO: Leave Time Out 10 folds [1] |
| Spatial | default parameters | spatial | LLO: Leave Location Out 10 folds [1] |
| Spatiotemporal | default parameters | Spatiotemporal | LTLO: Leave Time and Location Out 10 folds [1] |
| Custom | default parameters | default | Custom stratified bootstrapping that only selects one FoI value for each of the serosurveys. Account for the temporal correlation inherited from a catalytic model used to obtain FoI estimates |

The Boosted Regression Tree (BRT) and Random Forest (RF) methods seemed unaffected by the resampling strategies, with only a marginal improvement in predictions (+/- 1%) (Table 2).

However, the custom resampling strategy showed substantially worse performance and signs of overfitting, with an extremely low Resample *R*^2^ when using both Machine Learning (ML) methods. This strategy had been developed for the Linear Model (LM) framework, but it performed poorly with the ML frameworks. ML seemed to suffer from a small number of observations selected at each iteration (n=76). However, ML was able to handle temporal correlation directly, by using specially designed spatiotemporal resampling methods. Thus, spatiotemporal resampling methods available within the ML framework can be a straightforward and efficient alternative to the stratified bootstrapping method used previously [2]. The number of folds used in the resampling method did not impact the results and was set at 10.

Table 2: Median performance of Boosted Regression Trees (BRT) and Random Forest (RF) models for different resampling methods using median FoI (MedFoI) to fit the models and default parameters

| Model Name | *R*^2^ | | Resample *R*^2^ | |
| --- | --- | --- | --- | --- |
|  |  |  |  |  |
|  | BRT | RF | BRT | RF |
| Random | 0.83 | 0.98 | 0.82 | 0.97 |
| Temporal | 0.84 | 0.98 | 0.83 | 0.98 |
| Spatial | 0.84 | 0.98 | 0.83 | 0.98 |
| Spatiotemporal | 0.84 | 0.98 | 0.83 | 0.98 |
| Custom | 0.50 | 0.66 | -3.11 | -1.27 |

BRT: Boosted Regression Trees; RF: Random Forest methods; *R*^2^ is calculated on the entire dataset while the Resample *R*^2^ is calculated on the test set at each resampling iteration.

- - 1. Hyperparameters tuning

ML hyperparameters define the structure and complexity of the models and can be optimised to better fit the requirements of the dataset. Hyperparameters are tuned before the model is trained and tested.

Here, to minimize the computational time, the tuning of the hyperparameters was performed on the Median FoI only (MedFoI approach) and the list of hyperparameters tuned is presented in Table 3.

Table 3: Hyperparameters tested for the Boosted Regression Trees (BRT) and Random Forest (RF) as described in the mlr3 framework (programming environment built on R to simplify the use of Machine Learning methods [3])

| mlr3 name | generic name | function | default value | values tested |
| --- | --- | --- | --- | --- |
| ***BRT:*** |  |  |  |  |
| interaction.depth | Depth of trees | Number of splits in each tree; also called the tree complexity., e.g. allows interactions between factors if set to 2. | 1 | 1,2 |
| n.minobsinnode | Minimum node size | Minimal number of observations in terminal nodes | 10 | 5-100 |
| n.trees | Number of trees | The total number of trees that will be included in the model | 100 | 1000-7000 |
| shrinkage | learning rate | Defines the pace at which the algorithm moves on the error surface | 0.001 | 0.001-0.01 |
| train.fraction | Training fraction | Defines the proportion of the dataset that is used for fitting; the remaining proportion is used to evaluate the performance of the model | 1 | 0.90-0.50 |
| ***RF:*** |  |  |  |  |
| ntree | Number of trees | Number of trees built | 500 | 5-1000 |
| nodesize | Minimum node size | Minimal number of observations in nodes | 5 | 1-100 |

BRT: Boosted Regression Trees; RF: Random Forest methods

The tuning process was applied in two steps. Firstly, a random search of 100 evaluations was implemented with a large set of values tested for each hyperparameter (Table 2). Second, the search window was narrowed around the best values found at the first step and a grid search was applied with 100 further evaluations.

- - 1. Results of tuning

Five and two hyperparameters were tuned for, respectively, BRT and RF models. Table 4 presents the hyperparameter values tested at each step of the process.

Table 4: Tuning process for ML frameworks’ hyperparameters

| R name | abbreviation | default value |  | values tested at step 1 | values tested at step 2 |
| --- | --- | --- | --- | --- | --- |
| *BRT*: |  |  |  |  |  |
| interaction.depth | dep | 1 |  | 1,2 | 2 |
| n.minobsinnode | n.ob | 10 |  | 5-100 | 5--25 |
| n.trees | n.tr | 100 |  | 1000-7000 | 3000-5000 |
| shrinkage | shr | 0.001 |  | 0.001-0.01 | 0.004-0.009 |
| train.fraction | t.fr | 1 |  | 0.90-0.50 | 0.75-0.90 |
| *RF:* |  |  |  |  |  |
| ntree |  | 500 |  | 5-2000 | 5-100 |
| nodesize |  | 5 |  | 1-100 | 1-30 |

The hyperparameters obtained from the tuning of the models (n.ob=5, n.tr=4556, shr=0.01, dep=2 and t.fr=0.82%) were used to obtain the final results.

For RF, the cross validation *R*^2^ (CV *R*^2^), calculated on the cross validation set, gradually improved but reached a plateau after 25 trees and 5 nodes size; therefore, these values were used to fit the model. Higher numbers of trees and nodes size led to substantially larger computational cost but with marginal impact on model performance.

# Supplementary References

1. Meyer H, Reudenbach C, Hengl T, Katurji M, Nauss T. Improving performance of spatio-temporal machine learning models using forward feature selection and target-oriented validation. Environ Model Softw. 2018 Mar 1;101:1–9.

2. Ledien J, Cucunubá ZM, Parra-Henao G, Rodríguez-Monguí E, Dobson AP, Basáñez MG, et al. Spatiotemporal variations in exposure: Chagas disease in Colombia as a case study. BMC Med Res Methodol. 2022 Jan 13;22(1):13.

3. Lang M, Binder M, Richter J, Schratz P, Pfisterer F, Coors S, et al. mlr3: A modern object-oriented machine learning framework in R. J Open Source Softw. 2019 Dec 11;4(44):1903.
