# Supplementary material for: Linear and Machine Learning modelling for spatiotemporal disease predictions: Force-of-Infection of Chagas disease
Source: PLoS Negl Trop Dis. 2022 Jul 19;16(7):e0010594. doi: 10.1371/journal.pntd.0010594 (PMC9337653; doi:10.1371/journal.pntd.0010594)
Supplement: S2 Appendix — (DOCX) [file pntd.0010594.s002.docx]

**Linear and Machine Learning Modelling for Spatiotemporal Disease Predictions: Force-of-Infection of Chagas Disease**

**Julia Ledien^1^, Zulma M. Cucunubá^2,3^, Gabriel Parra-Henao^4,5^, Eliana Rodríguez-Monguí^6^, Andrew P. Dobson^7^, Susana B. Adamo^8^, María-Gloria Basáñez^2^, Pierre Nouvellet^1^**

¹School of Life Sciences, University of Sussex, Falmer, Brighton, UK

²London Centre for Neglected Tropical Disease Research & MRC Centre for Global Infectious Disease Analysis, School of Public Health, Imperial College London, London, UK

^3^Departamento de Epidemiología Clínica y Bioestadística, Facultad de Medicina, Universidad Pontificia Javeriana, Bogotá, Colombia

^4^Centro de Investigación en Salud para el Trópico, Universidad Cooperativa de Colombia, Santa Marta, Colombia

^5^National Institute of Health, Bogotá, Colombia

^6^ Independent consultant to the Neglected, Tropical and Vector Borne Diseases Program, Pan American Health Organization (PAHO), Colombia

^7^Department of Ecology and Evolutionary Biology, Princeton University, Princeton, New Jersey, USA

^8^Center for International Earth Science Information Network (CIESIN), The Earth Institute, Columbia Climate School, Columbia University, New York, USA

Corresponding author: Julia Ledien, School of Life Sciences, University of Sussex, UK, [j.ledien@sussex.ac.uk](mailto:j.ledien@sussex.ac.uk)

**S2 Appendix: Comparing observations and predictions across serosurveys (extracted from** [1]**)**

“For each serosurvey, we compared, across years, the median and 95%CI (Confidence Interval) of the predicted FoI against the median and 95%CrI (Credible Interval) of the originally estimated FoI [2] (i.e. the dependent variable or ‘observed’ FoI).

For each quantile of interest $q_{x}$ (i.e., median, 2.5%, and 97.5% percentiles, denoted $q_{m}$, $q_{l}$ and $q_{u}$ respectively), we computed a distance between the ‘observed’ and predicted quantile ($\delta_{q_{x}}$). This distance was standardised by the interval between the observed median and observed upper (or lower) 95% CrI,

| $\left\{ \begin{matrix} \delta_{q_{x}}=\frac{q_{x}\left( \hat{y} \right)-q_{x}\left( y \right)}{q_{x}\left( y \right)-q_{l}\left( y \right)} & if q_{x}\left( \hat{y} \right)<q_{x}\left( y \right) \\ \delta_{q_{x}}=\frac{q_{x}\left( \hat{y} \right)-q_{x}\left( y \right)}{q_{u}\left( y \right)-q_{x}\left( y \right)} & if q_{x}\left( \hat{y} \right)>q_{x}\left( y \right) \end{matrix} \right.$ | (Eq. 3) |
| --- | --- |

When the predicted and ‘observed’ medians are equal, we expect $\delta_{q_{m}}=0$. If the predicted median was equal to the upper (or lower) 95%CrI of the ‘observed’ FoI values, then we would have $\delta_{q_{m}}=1$ ($\delta_{q_{m}}=-1$).

If the predicted and ‘observed’ upper (or lower) 95% CI/CrI were equal, then we would expect $\delta_{q_{u}}=1$ ($\delta_{q_{u}}=-1$). A value $\delta_{q_{u}}=2$ would indicate that the interval between the median and upper CI in the prediction is twice as wide as the interval between the median and upper CrI in the observations.

The change in the denominator reflects the non-symmetrical nature of the 95%CI.

As it is rescaled, this measure of bias allows an assessment of the predictive ability of our modelling approaches across serosurveys. For each year, we estimated the median and interquartile range in the bias. This was also done by setting.

Supplementary References

1. Ledien J, Cucunubá ZM, Parra-Henao G, Rodríguez-Monguí E, Dobson AP, Basáñez MG, et al. Spatiotemporal variations in exposure: Chagas disease in Colombia as a case study. BMC Med Res Methodol. 2022 Jan 13;22(1):13.

2. Cucunubá ZM, Nouvellet P, Conteh L, Vera MJ, Angulo VM, Dib JC, et al. Modelling historical changes in the force-of-infection of Chagas disease to inform control and elimination programmes: application in Colombia. BMJ Glob Health. 2017;2(3):e000345.
