# Supplementary material for: Linear and Machine Learning modelling for spatiotemporal disease predictions: Force-of-Infection of Chagas disease
Source: PLoS Negl Trop Dis. 2022 Jul 19;16(7):e0010594. doi: 10.1371/journal.pntd.0010594 (PMC9337653; doi:10.1371/journal.pntd.0010594)
Supplement: S1 Table — (DOCX) [file pntd.0010594.s003.docx]

**Linear and Machine Learning Modelling for Spatiotemporal Disease Predictions: Force-of-Infection of Chagas Disease**

**Julia Ledien^1^, Zulma M. Cucunubá^2,3^, Gabriel Parra-Henao^4,5^, Eliana Rodríguez-Monguí^6^, Andrew P. Dobson^7^, Susana B. Adamo^8^, María-Gloria Basáñez^2^, Pierre Nouvellet^1^**

¹School of Life Sciences, University of Sussex, Falmer, Brighton, UK

²London Centre for Neglected Tropical Disease Research & MRC Centre for Global Infectious Disease Analysis, School of Public Health, Imperial College London, London, UK

^3^Departamento de Epidemiología Clínica y Bioestadística, Facultad de Medicina, Universidad Pontificia Javeriana, Bogotá, Colombia

^4^Centro de Investigación en Salud para el Trópico, Universidad Cooperativa de Colombia, Santa Marta, Colombia

^5^National Institute of Health, Bogotá, Colombia

^6^ Independent consultant to the Neglected, Tropical and Vector Borne Diseases Program, Pan American Health Organization (PAHO), Colombia

^7^Department of Ecology and Evolutionary Biology, Princeton University, Princeton, New Jersey, USA

^8^Center for International Earth Science Information Network (CIESIN), The Earth Institute, Columbia Climate School, Columbia University, New York, USA

Corresponding author: Julia Ledien, School of Life Sciences, University of Sussex, UK, [j.ledien@sussex.ac.uk](mailto:j.ledien@sussex.ac.uk)

**S1_Table: Variables tested as factors in the geospatial analyses of Chagas disease in Colombia**

| Name | Description | Spatial scale | Data Source |
| --- | --- | --- | --- |
| **Serosurvey characteristics:** |  |  |  |
| Year of the survey | Year when the serosurvey was conducted | ‒ | [1] |
| Setting | Setting of the serosurvey: urban, rural, indigenous or mixed | ‒ | [1] |
| Latitude | latitude of the centroid of the catchment area of the serosurvey | ‒ | [1] |
| Longitude | longitude of the catchment area of the serosurvey | ‒ | [1] |
| **Blood banks data:** |  |  |  |
| Seroprevalence | Number of blood units positive for *Trypanosoma cruzi* divided by the number of blood units tested. Data aggregated for 1993–2010 by department | Department | PAHO |
| Proportion of blood units screened | Number of blood units tested for *T. cruzi* divided by the number of blood units received. Data aggregated for 1993–2010 by department | Department | PAHO |
| **Demography:** |  |  |  |
| Population density | Estimates of the annual population size at municipality level from the government divided by the surface of the municipality in km^2^ extracted from GDAM shapefiles | Municipality | [2] |
| Poverty | Proportion of households with deficit from 1993 census for 1950‒1999 and from 2005 census for 2000‒2014 | Municipality | [2] |
| Rural Indigenous Population size | Population size of the indigenous communities living in rural areas from 2005 census | Department | [2] |
| **Climate:** |  |  |  |
| *Continuous* |  |  |  |
| Polar climate frequency | Number of pixels defined as polar climate divided by the total number of pixels in the municipality | Municipality | [3] |
| Tropical climate frequency | Number of pixels defined as tropical climate divided by the total number of pixels in the municipality | Municipality | [3] |
| Temperate climate frequency | Number of pixels defined as temperate climate divided by the total number of pixels in the municipality | Municipality | [3] |
| Arid climate frequency | Number of pixels defined as arid climate divided by the total number of pixels in the municipality | Municipality | [3] |
| *Categorical* |  |  |  |
| Tropical climate categorized | Tropical climate frequency categorized as follows: low (<10%), medium (10%‒60%), large (60%‒90%) and extra‒large (>90%) | Municipality | [3] |
| **Entomological data:** |  |  |  |
| *At Departmental level* |  |  |  |
| *R.* *prolixus* geographical extent | Number of municipalities where *Rhodnius* *prolixus* is present divided by the number of municipalities in the department. Combined data from National report of 2013 and more recent data from Parra-Henao *et al*. | Department | [4–6] |
| *T. dimidiata* geographical extent | Number of municipalities where *Triatoma* *dimidiata* is present divided by the number of municipalities in the department. Combined data from National report of 2013 and more recent data from Parra-Henao *et al.* | Department | [4–6] |
| *R.* *prolixus* presence | Presence of *R.* *prolixus* in the department (yes/no). Combined data from National report of 2013 and more recent data from Parra-Henao *et al*. | Department | [4–6] |
| *T*. *dimidiata* presence | Presence of *T.* *dimidiata* in the department (yes/no). Combined data from National report of 2013 and more recent data from Parra-Henao *et al.* | Department | [4–6] |
| *At Municipality level* |  |  |  |
| *R.* *prolixus* density | Number of *R.* *prolixus* specimens found divided by the number of households in the municipality. Data extracted from the National report of 2013 | Municipality | [4] |
| *T.* *dimidiata* density | Number of *T.* *dimidiata* specimens found divided by the number of households in the municipality. Data extracted from the National report of 2013 | Municipality | [4] |
| *R. prolixus* presence | Presence of *R. prolixus* in the municipality (yes/no). Combined data from National report of 2013 and more recent data from Parra-Henao *et al*. | Municipality | [4–6] |
| *T. dimidiata* presence | Presence of *T. dimidiata* in the municipality (yes/no). Combined data from National report of 2013 and more recent data from Parra-Henao *et al*. | Municipality | [4–6] |
| **Interventions:** |  |  |  |
| *At Municipality level* |  |  |  |
| Intervention intensity | Number of municipalities where interventions were organized divided by the number of municipalities in the department for the following time periods: before 1996, 1996‒2000, 2001‒2010 and 2011‒2014. | Department | [7] |
| Intervention category | Municipality-level intervention intensity categorized as follows: no intervention (0%), low (0%‒25%), medium (25%‒50%), high (50%‒75%), very high (>75%) | Department | [7] |
| *At Household level* |  |  |  |
| Household intervention | Number of households having received interventions divided by the total number of households in the department for the following time periods: before 1996, 1996‒2000, 2001‒2010 and 2011‒2014 | Department | [7] |
| Household intervention category | Household-level intervention intensity categorized as follows: no intervention (0%), medium (0%‒10%), high (>10%) | Department | [7] |
| **Time:** |  |  |  |
| Year | Year of the FoI value | ‒ | ‒ |
| Decade | Decade of the FoI value defined as follows: 1[1900‒1909], 2[1910‒1919], 3[1920‒1929], 4[1930‒1939], 5[1940‒1949], 6[1950‒1959], 7[1960‒1969], 8[1970‒1979], 9[1980‒1989], 10[1990‒1999], 11[2000‒2009], 12[2010‒2014] | ‒ | ‒ |

# Supplementary References

1. Cucunubá ZM, Nouvellet P, Conteh L, Vera MJ, Angulo VM, Dib JC, et al. Modelling historical changes in the force-of-infection of Chagas disease to inform control and elimination programmes: application in Colombia. BMJ Glob Health. 2017;2(3):e000345.

2. Departamento Administrativo Nacional de Estadística (DANE): www.dane.gov.co [Internet]. [cited 2022 Jan 25]. Available from: https://www.dane.gov.co/index.php/estadisticas-por-tema/demografia-y-poblacion

3. Beck HE, Zimmermann NE, McVicar TR, Vergopolan N, Berg A, Wood EF. Present and future Köppen-Geiger climate classification maps at 1-km resolution. Sci Data. 2018 Oct 30;5:180214.

4. Parra-Henao GJ, Flórez Martínez M, Angulo Silva VM. Vigilancia de Triatominae (Hemiptera: Reduviidae) en Colombia. In: Red Chagas Colombia. 1era Edition., p. 127. Bucaramanga Colombia: Sic Editorial Ltda; 2013. [Memorias del Curso de Capacitación Métodos Básicos en Epidemiología y Redacción Científica. Septiembre 23 a 27 de 2013. Bogotá D.C. Colombia.].

5. Parra-Henao G, Quirós-Gómez O, Jaramillo-O N, Segura-Cardona Á. Environmental determinants of the distribution of Chagas disease vector Triatoma dimidiata in Colombia. Am J Trop Med Hyg. 2016 Apr;94(4):767–74.

6. Parra-Henao G, Suárez-Escudero LC, González-Caro S. Potential distribution of Chagas disease vectors (Hemiptera, Reduviidae, Triatominae) in Colombia, based on ecological niche modeling. J Trop Med. 2016;2016:1439090.

7. Parra-Henao G, Angulo V, Cucunubá Z. Colombian Chagas Network. Final report, project 1. 2015.
