# Supplementary figures and images for: Linear and Machine Learning modelling for spatiotemporal disease predictions: Force-of-Infection of Chagas disease
Source: PLoS Negl Trop Dis. 2022 Jul 19;16(7):e0010594. doi: 10.1371/journal.pntd.0010594 (PMC9337653; doi:10.1371/journal.pntd.0010594)

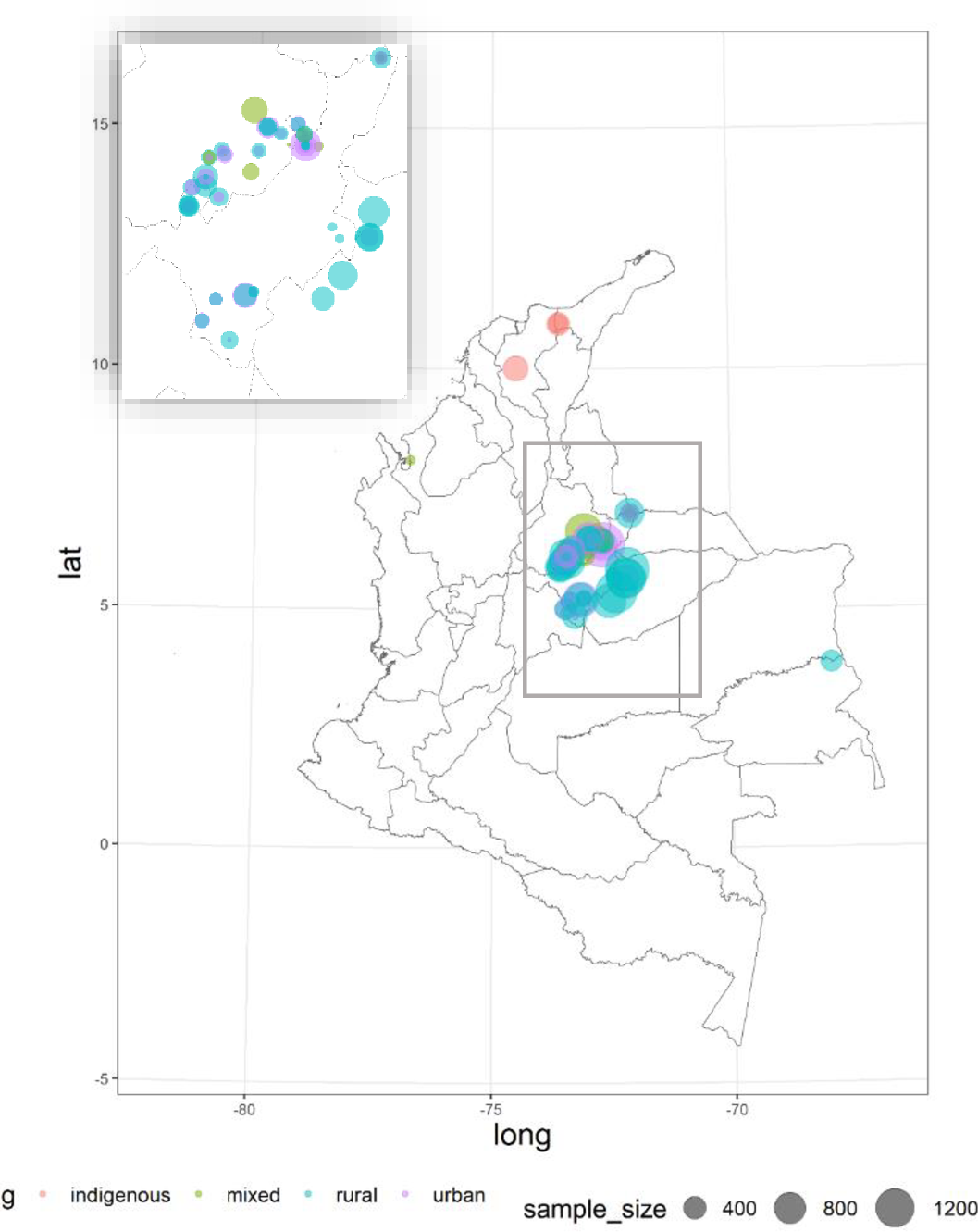

Supplement: S1 Fig — Figure extracted from (2). The grey boundaries delimitate the Departments. (TIF) [file pntd.0010594.s004.tif]

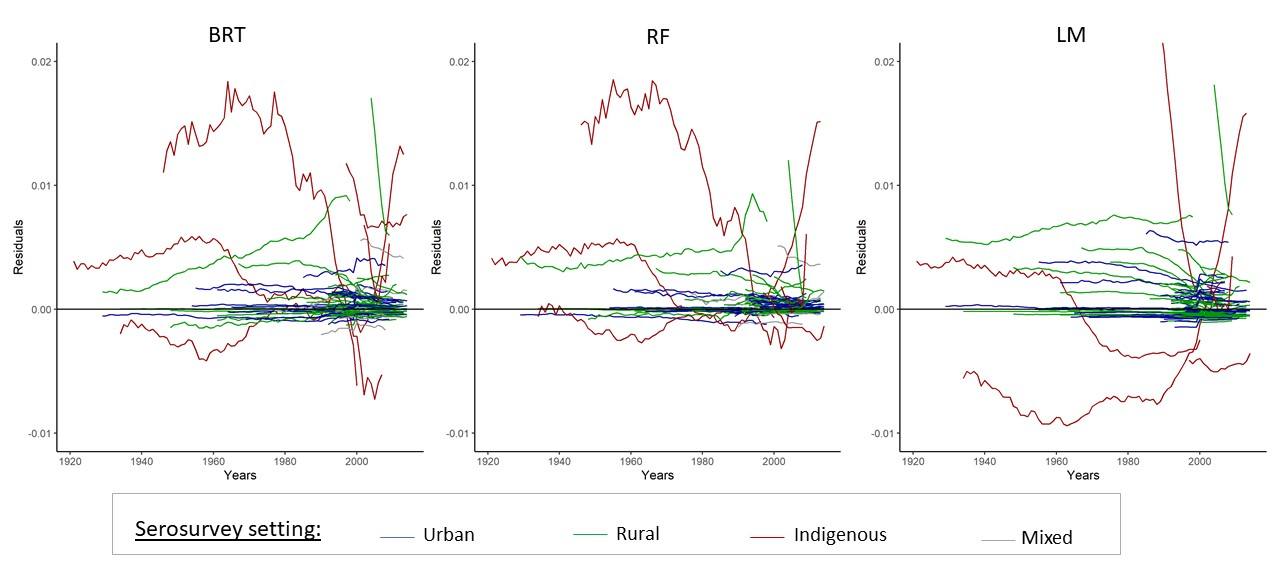

Supplement: S2 Fig — Each line corresponds to one serosurvey. (TIF) [file pntd.0010594.s005.tif]
