# Supplementary material for: Linear and Machine Learning modelling for spatiotemporal disease predictions: Force-of-Infection of Chagas disease
Source: PLoS Negl Trop Dis. 2022 Jul 19;16(7):e0010594. doi: 10.1371/journal.pntd.0010594 (PMC9337653; doi:10.1371/journal.pntd.0010594)
Supplement: Attachment — Submitted filename: Point-by-point response to reviewers.docx [file pntd.0010594.s006.docx]

Response to reviewers’ comments

Submission ID PNTD-D-22-00186

Title: Linear and Machine Learning Modelling for Spatiotemporal Disease Predictions: Force-of-Infection of Chagas Disease

Dear editor,

We revised our manuscript and addressed all reviewers’ comments. In particular, we have added the scripts to our repository folder and clarified some aspects of the methodology.

The line numbers provided in this document refer to the paper submitted with the track changes.

Reviewer #1:

Comment 1.1

**This work compared the ability of machine learning algorithms (here: random forest and boosted regression trees) and linear models to quantify the uncertainty around central estimates such as means or medians. The authors further compared the performance of all three modelling frameworks and their ability to predict the force of infection of Chagas disease across space and time. They have used robust age-stratified serological data from Colombia as well as relevant remotely-sensed covariates to perform all analyses. The work concluded that machine learning algorithms out-performed linear models in capturing accurate estimates for central tendency. However, linear models provided more robust estimates of uncertainty.**

**This work is particularly important given the recent popularity and use of machine learning algorithms in medicine research and disease prediction. The Objectives of the study were clearly articulated and are relevant in the current sphere of disease modelling methodologies. The Methods and Results were appropriate without any ambiguities. Similar sets of data were used in all three modelling frameworks, allowing for a more complete comparison. Results were complete with ample information to aid unbiased interpretation. Authors provided plentiful important details, and often overlooked information, such as those provided in Table 5. I found this interesting. Discussion/Conclusions was balanced. This is an all-round excellent paper. I recommend that it can be published as it is, without any revision.**

Authors’ reply

We thank the reviewer for praising our manuscript and highlighting its methodological rigor..

Reviewer #2:

Comment 2.1

**The Github repository may be private? It's not accessible using the link provided. The only repository associated with the user does not contain analysis scripts.**

Authors’ reply

We thank the reviewer for his interest in the manuscript and the data and method employed. The repository, indeed, only contained the data used for the analyses and not the scripts. As suggested, a new folder within this repository have been added to include the scripts of the analyses.

**How were correlated variables dealt with? Particularly with LM?**

Authors’ reply

We thank the reviewer for his comment. Within the Machine Learning framework correlation between covariate does not cause analytical issue so it has been ignored. Within the Linear model framework, each linear model is composed of a set of predictors that are uncorrelated. More detail about the Linear Model is available in a previous publication. We have chosen to limit the information provided about Linear Models in this paper to avoid having supplementary material that would be too long and repetive with our previous work.

To clarify we added l134:

“The LM framework relied on a list of plausible linear combinations of predictors **selected based on expert knowledge and to avoid correlation between predictors. They** were then integrated into an ensemble model using model averaging with weights based on the performance indicators of each individual linear model.”

Also, we added l212:

“While the LM framework relies on a list of plausible and pre-defined linear models including interactions between factors (predictors) **and excluding correlated predictors**, the ML framework is implemented in two steps, to be fitted only on the ten most important variables..”

**How were training and testing datasets chosen? Were these random or stratified across space and time?**

Authors’ reply

As described in the method section, a spatiotemporal resampling have been applied for the ML methods. The details have been provided in supplement. Regarding the LM, a random selection have been applied and is described in supplement and in another paper available here ( DOI: [10.1186/s12874-021-01477-6](https://doi.org/10.1186/s12874-021-01477-6) ).

To clarify we added l218:

“Finally, ML requires a tuning step, during which the best hyperparameters are selected. **Also, the resampling strategy used within the LM framework relies on random resampling while a spatiotemporal resampling strategy has been used for the ML framework.** A detailed description of the tuning of hyperparameters and the comparison of several resampling strategies is available in S1 Appendix of the Supporting Information File, including details about the tuning of hyperparameters and the comparison of several resampling strategies.”

Comment 2.2

**Can the discussion of the results discuss**

**1) Model performance for median FOI predictions. Also including a discussion of different predictors and how the direction and magnitude of these predictors varied between models.**

**2) Then discuss the overlap with posterior (ability to capture uncertainity)**

**Now it jumps between evaluation metrics, response variables and models and is difficult to follow. If this section is more linked to specific research questions, I think it would be easier to follow**

Authors’ reply

The performance of the median FoI and overlap are presented in the Table 1 and are discussed from the line 262 of the results section.

The Table 2 shows the predictors included for each method and their importance/magnitude. The direction of the predictors is not available directly for Machine Learning methods as non-linear relations are allowed (the associations are based on threasold). Regarding the Linear Model framework, the direction of the predictors are presented in the Figure 5 of our previous paper available here ( DOI: [10.1186/s12874-021-01477-6](https://doi.org/10.1186/s12874-021-01477-6) ).

Comment 2.3

**What are the key outcomes of this research?**

**How applicable is this to other settings? Presumably without FOI estimates from age seroprevalence data, this would be very difficult to do.**

Authors’ reply

The key outcome of this research is the emphasis on the necessity to use the entire uncertainty on the Force-of-Infection when using a predictive model whatever the method used is. In addition, the modelling method (LM or ML) chosen need to be selected according to the objectives of the analyses as different frameworks have different pros and cons.

We added l471:

“We show that **the uncertainty on the input variables cannot be neglected whatever statistical method is used and furthermore that** the choice of modelling framework requires careful consideration according to the ultimate objectives of the modelling endeavour..”

We agree with the reviewer in stating that ‘without FOI estimates from age seroprevalence data, this would be very difficult to do’. We have stressed in the abstract and in the conclusions that:

‘Our approach can be extended to the modelling of FoI patterns […] to other infectious diseases for which serosurveys are regularly conducted for surveillance.’

And

‘… the modelling frameworks compared here can be applied […] to infectious diseases (including neglected tropical diseases) for which age-stratified serological data are regularly collected.’

Comment 2.4

**This paper compares the ability of ML (RF & BRT) and LM to identify key predictors of T. cruzi FOI over space and time in Colombia. Observed FOI estimates come from previous work conducted by some of the research team and used age-seroprevalence data to estimate FOI by year and municipality. Both the median FOI estimate and the full posterior distribution were used as response variables in LM and ML models.**

**While it is clear a lot of work went into this paper, it’s very difficult to follow in its current form. More consistent terms throughout should help the reader follow which methods and which tests are used. For example, “FoI estimates” is used to describe the previous age-seroprevalence estimates but also the LM and ML predictions. Using these interchangeably creates unnecessary confusion and makes the key findings difficult to disentangle from previous work.**

Authors’ reply

We would like to thanks the reviewer for her/his comments and confirm that we have check the consistency of the terms we have used. The manuscript also includes a list of abbreviations to make the reading very clear (see l488).

Reviewer #3:

Comment 3.1

**The aim is to compare the performance of two Machine Model frameworks (Bosted Regression Tree - BRT; Random Forest - RF) with a Linear Model regression.**

**From the theoretical field, it is well designed and explained. The analysis is well established and methodologically corresponds to the objectives.**

**It is observed that the study population results from historical data and grouped at the community level, for which there is a bias in estimating that the risk of infection is distributed randomly when the result of the new infection results from environmental conditions. (housing, environmental interventions and insecticide use), of the person in terms of exposure (influenced by age, socioeconomic status and activity), vector (density, location, etc.) and parasites (density and dispersion)**

**It is expected that this situation will produce bias in the final results.**

**The presentation of the results is adequate and they are shown clearly.**

**The conclusions adequately inform the data presented, informing the limitations of the analysis as well as the diverse conditions that are presented.**

**The authors present their data and propose the continuation of analyzes of this type.**

**The authors adequately express the objective of comparing the performance of two Machine Model frameworks, the results are adequately presented and in the discussion they address the different topics, clearly establishing the limitations and application of what is proposed.**

Authors’ reply

We thank the reviewer for his/her comments.
